# Mini-Implants in the Anchorage Armamentarium: New Paradigms in the Orthodontics

**DOI:** 10.1155/2012/394121

**Published:** 2012-06-05

**Authors:** Masaru Yamaguchi, Toshihiro Inami, Ko Ito, Kazutaka Kasai, Yasuhiro Tanimoto

**Affiliations:** ^1^Department of Orthodontics, Nihon University School of Dentistry at Matsudo, 2-870-1 Sakaecho-Nishi, Chiba, Matsudo 271-8587, Japan; ^2^Maxillofacial Surgery, Nihon University School of Dentistry at Matsudo, Chiba, Matsudo 271-8587, Japan; ^3^Dental Biomaterials, Nihon University School of Dentistry at Matsudo, Chiba, Matsudo 271-8587, Japan

## Abstract

Paradigms have started to shift in the orthodontic world since the introduction of mini-implants in the anchorage armamentarium. Various forms of skeletal anchorage, including miniscrews and miniplates, have been reported in the literature. Recently, great emphasis has been placed on the miniscrew type of temporary anchorage device (TAD). These devices are small, are implanted with a relatively simple surgical procedure, and increase the potential for better orthodontic results. Therefore, miniscrews not only free orthodontists from anchorage-demanding cases, but they also enable clinicians to have good control over tooth movement in 3 dimensions. The miniplate type also produces significant improvements in treatment outcomes and has widened the spectrum of orthodontics. The purpose of this paper is to update clinicians on the current concepts and versatile uses and clinical applications of skeletal anchorage in orthodontics.

## 1. Introduction

The goal of orthodontic treatment is to improve the patient's life through enhancement of dentofacial functions and esthetics. Anchorage, defined as a resistance to unwanted tooth movement [1], is a prerequisite for the orthodontic treatment of dental and skeletal malocclusions [2, 3]. 

Controlling anchorage helps to avoid undesirable tooth movements. However, even a small reactive force can cause undesirable movements; it is important to have absolute anchorage to avoid them [4, 5]. Absolute or infinite anchorage is defined as no movement of the anchorage unit (zero anchorage loss) as a consequence to the reaction forces applied to move teeth [1]. Such an anchorage can only be obtained by using ankylosed teeth or dental implants as anchors, both relying on bone to inhibit movement [6]. Anchorage provided by devices, such as implants or miniscrew implants fixed to bone, may be obtained by enhancing the support to the reactive unit (indirect anchorage) or by fixing the anchor units (direct anchorage), thus facilitating skeletal anchorage.

Orthodontic anchorage is an important factor in obtaining good treatment results. Stable anchorage is a pre-requisite for orthodontic treatment with fixed appliances. Traditional appliances for reinforcement of anchorage have included headgear and intraoral elastics. The inclusion of implants for skeletal anchorage can move a tooth without the use of headgear and intraoral elastics.

Skeletal anchorage with temporary anchorage devices (TADs) has been widely incorporated into orthodontic treatment for expanding the boundary of tooth movement without patient compliance [7–10]. TAD skeletal anchorage is especially useful for treating malocclusion with vertical problems such as open bite and overeruption of teeth due to loss of antagonists [11–17]. Traditionally, skeletal open bite requires aggressive surgical impaction to reduce the maxillary dentoalveolar height. Supererupted teeth were usually corrected by endodontic intervention and crown restoration at the expense of tooth vitality before TAD skeletal anchorage became popular. However, orthodontic intrusion with TAD skeletal anchorage provides a conservative treatment approach with little irreversible damage if patients can accept a longer treatment time [11, 12, 16, 17]. TAD skeletal anchorage is not only useful for resolving vertical problems in orthodontics but also eliminates the need for patient compliance for sagittal dental movement such as mesializing or distalizing the entire dentition both with and without extraction [10, 18]. With a correct diagnosis and mechanical design, TAD skeletal anchorage is sufficiently versatile to treat all types of malocclusions, except those accompanied by facial deformities requiring invasive and extensive surgeries to obtain a harmonious skeletal relationship [18, 19].

Various types of TAD have been used in orthodontics [20, 21]. Turley et al. [2] and Roberts et al. [22]reported conventional osseointegrated implants. Costa et al. [7] and Freudenthaler et al. [23] reported mini- and microimplants and Wehrbein et al. [24–26] reported palatal implants.

The aim of this paper is to present the development, clinical use, benefits, and drawbacks of the miniscrew and plate type implants used to obtain a temporary but absolute skeletal anchorage for orthodontic applications.

## 2. Dental Implant and Mini-Implants

Titanium implants have been used largely in dentistry over past decades. The close contact between bone and titanium implants provides an ankylosis-like type of interaction, an event named osseointegration [27]. Because osseointegration offers necessary conditions for load and transfer bearing, the use of dental implants as orthodontic anchorages has increased progressively over the years [22, 28]. Although implants provide excellent anchorage, some limitations such as the waiting time for allowing osseointegration, invasive surgery, high cost, and difficulty of removing the dental implant after completion of orthodontic treatment were noted initially because of their routine use in orthodontics [29, 30]. Another initial difficulty was that conventional implants are placed in edentulous sites with sufficient bone for anchorage; however, most orthodontic patients are young and do not have edentulous areas. To overcome this limitation, titanium screws with smaller dimensions (miniscrews) were introduced and were referred to as orthodontic mini-implants [31]; these can be placed in unconventional sites such as the alveolar bone of adjacent teeth without damaging roots and without requiring time for osseointegration [32–34]. Furthermore, Rinaldi and Arana-Chavez showed that repair occurred at the mini-implant surface through cementoblastic activity. In addition, the periodontal ligament space was well preserved in all specimens, and no microankylotic spots were detected [35]. 

## 3. Two Main Systems

**Figure 1:**
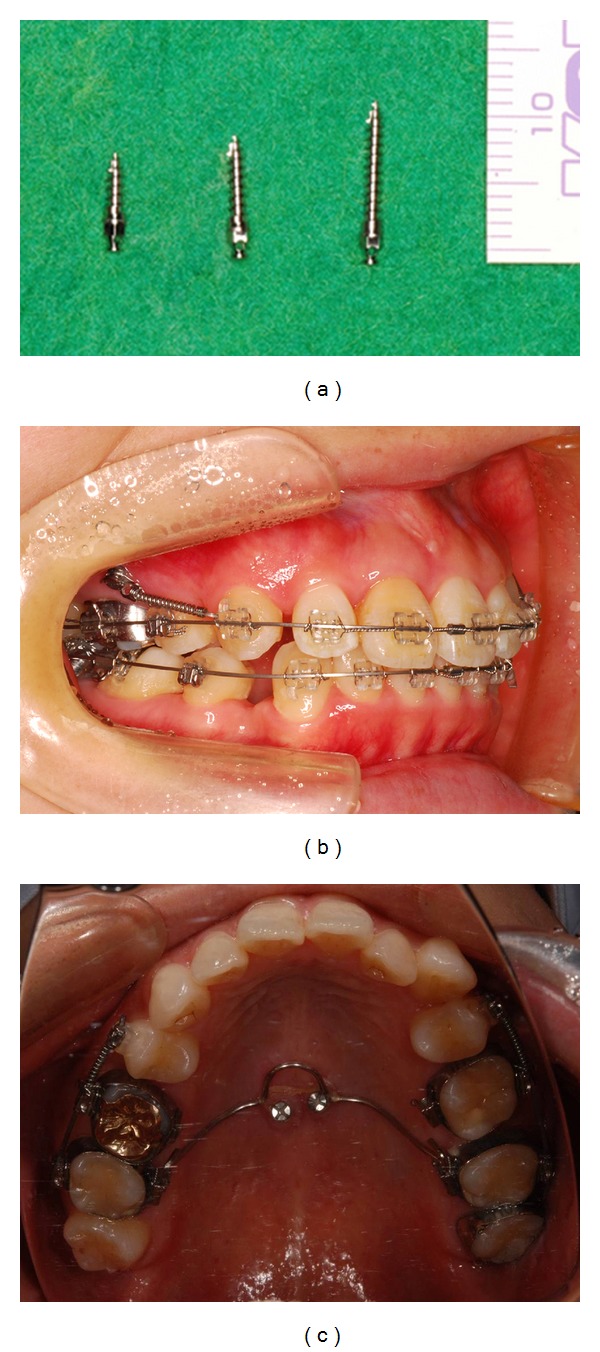
(a) Miniscrews, (b) the maxillary right canine was retracted with a closed coil from miniscrew, and (c) palatal implants. The maxillary 2nd molars were connected with trance palatal arch and palatal implants.

**Figure 2:**
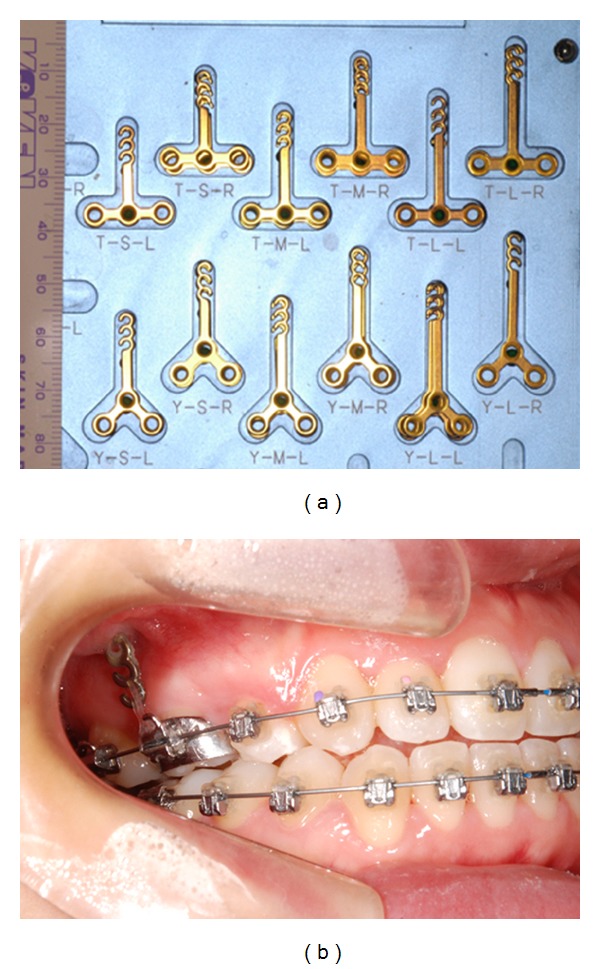
(a) Miniplates, (b) the maxillary right 1st molar was intruded with an elastic chain from miniplate.

Two main systems are used to retract the anterior teeth: miniscrews (Figures 1 and 2) [36–44] and miniplates [45, 46].

### 3.1. Miniscrews

#### 3.1.1. Palatal Implants

Most of the published studies on the retraction of anterior teeth with miniscrews are case reports [29, 41–44] (Figure 1(a)). In the cases presented, the miniscrews were applied directly to the hooks on the archwire to retract all upper 6 anterior teeth simultaneously with a loading force of about 150 g. Furthermore, the extraction space was fully utilized in the retraction of anterior teeth without anchorage loss. The posterior teeth even moved distally slightly in some cases [41–44].One of the advantages of the mechanics involved in these cases was the direct application of load to the vertical hooks on the archwire: in this setup, the point of force application was close to the center of resistance of the anterior segment, thereby allowing bodily sliding of the whole segment with minimal tipping, and in turn, shortening the treatment time [44] (Figure 1(b)). 

In the cases inserted within palatal, Wehrbein et al. [24] prospectively studied 9 patients with Class II malocclusion in whom anchorage was indirectly reinforced by connection of a transpalatal bar to a palatal implant after extraction of the upper first premolars. The loading force applied was 200 g over 11 months, and the reduction of overjet ranged from 5.1 to 7.8 mm (mean, 6.22 mm). The loss of anchorage ranged from 0.2 to 1.6 mm, and was attributed to the deformation of the transpalatal bar (Figure 1(c)). 

### 3.2. Miniplates

In 1985, Jenner and Fitzpatrick [47] reported an alternative orthodontic anchorage method using a bone plate. Umemori et al. [11] introduced miniplate skeletal anchorage that was effective in controlling the cant and level of the occlusal plane during orthodontic open-bite correction without serious side-effects. Rattanayatikul et al. [48] described the use of miniplates for temporary skeletal anchorage in treating skeletal Class III malocclusions with missing posterior teeth. Tseng et al. [49] reported that miniplates as skeletal anchorage are effective for managing severely impacted mandibular second molars.

Miniplates have also been used to retract anterior teeth [45, 46]. De Clerck et al. [45] followed up 27 patients undergoing retraction of canines (11 bilateral and 16 unilateral) using a miniplate fixed with 3 miniscrews. The setup used sliding mechanics with power arms attached to the canines and a loading force of 50 to 100 g. The mean rate of distalization among the patients studied was 1.14 mm per month. 

The miniplate's one end is fixed to the infrazygomatic crest and the other end has attachments to engage orthodontic auxiliaries. Meanwhile, the miniscrew is fixed to only the alveolar cortical bone. Therefore, higher loading rate should be applicable to miniplates rather than miniscrews as the direct bone anchor is available in case of miniplates. 

## 4. Tooth Movement

### 4.1. Retraction of Anterior Teeth

Park et al. [50] described a case of anterior retraction in which an innovative miniscrew technique circumvented the need for brackets during retraction. First, maxillary miniscrews were placed between the first molar and second premolar. Second, a segmental hard acrylic splint with 2 lever arms distal to the canines was fabricated on the 6 anterior teeth. Elastics were then attached from the miniscrews to the lever arm. The 6 anterior teeth that were embedded in the clear splint were thus retracted without a bracket during the 6 months of retraction. Brackets were needed only in the finishing stage in the last 6 months. In a prospective split-mouth study, Thiruvenkatachari et al. [51] measured anchorage loss during canine retraction in 10 patients in whom only 1 side of the mouth received miniscrew treatment. The canines were retracted in 4 to 6 months, with no anchorage loss on the implant side but with 1 to 2 mm of anchorage loss on the nonimplant side.

### 4.2. Intrusion of Dentition

Intrusion of posterior or anterior dentition is always difficult to achieve without the side effect of extrusion of the anchorage teeth, and the placement of mini-implants for skeletal anchorage may provide the solution. For example, intrusion of posterior teeth is essential in the correction of open bite, and case reports have shown that miniplates can lead to the intrusion of upper and lower molars by 3 to 5 mm, while also achieving counterclockwise mandibular rotation [52–55]. Sugawara et al. [56] investigated the amount of intrusion of mandibular molars among 9 patients after miniplate treatment, and found that 1.7 mm and 2.8 mm of intrusion was achieved in first and second molars, respectively, although there was about 30% relapse. Erverdi et al. [12] also reported using miniplates to intrude upper molars by 2.6 mm in 10 patients. Even as early as 1983, Creekmore and Eklund [57] demonstrated the use of miniscrews to intrude maxillary central incisors by 6 mm. In 2005, Ohnishi et al. [58] described a case of gummy smile correction with intrusion of the upper incisors by 3.5 mm. 

### 4.3. Intrusion or Extrusion of Individual Teeth

In the management of overeruption of unopposed teeth, molar intrusion is a common indication for orthodontic treatment before prosthodontic replacement of missing teeth. Two cases have been reported in which overerupted lower and upper molars were intruded with miniscrews but without any braces on other teeth [59, 60]. Upper molars can also be intruded with miniscrews on buccal and palatal sides before the prosthetic restoration of the lower missing teeth is commenced [61, 62]. In another case, overerupted upper left first and second molars were intruded by the fixation of a miniplate on buccal bone and a miniscrew on palatal bone, with a loading force of 150 to 200 g delivered by a power chain [17].

A miniscrew has been used for forced tooth extrusion in a 51-year-old woman who presented with a bridge that replaced a missing upper right incisor with the central incisor and canine as abutments. Because the gingiva at the central incisor and canine had receded by 3 to 4 mm, both of them required extrusion to match the gingival level of the contralateral side before a new bridge could be constructed. To do this, a miniscrew was placed into the alveolus of the missing upper lateral incisor and an open coil was applied perpendicularly to an orthodontic wire connecting the central incisor and canine [63].

## 5. Complications

Kravitz and Kusnoto [64], reviewed the potential risks and complications of orthodontic miniscrews with regard to insertion, orthodontic loading, and peri-implant soft tissue health.

### 5.1. Trauma to the Periodontal Ligament or the Dental Root during Insertion

Interradicular placement of orthodontic miniscrews risks trauma to the periodontal ligament or the dental root. Potential complications of root injury include loss of tooth vitality, osteosclerosis, and dentoalveolar ankylosis [65, 66]. Trauma to the outer dental root without pulpal involvement will most likely not influence the tooth's prognosis [67]. Dental roots damaged by orthodontic miniscrews have demonstrated complete repair of tooth and periodontium in 12 to 18 weeks after removal of the miniscrew. Interradicular placement requires proper radiographic planning, including surgical guide with panoramic and periapical radiographs to determine the safest site for miniscrew placement [29, 32, 68–70]. In the maxillary buccal region, the greatest amount of interradicular bone is between the second premolar and the first molar, 5 to 8 mm from the alveolar crest [71–73]. In the mandibular buccal region, the greatest amount of interradicular bone is either between the second premolar and the first molar, or between the first molar and the second molar, approximately 11 mm from the alveolar crest [71–73]. During interradicular placement in the posterior region, there is a tendency for the clinician to change the angle of insertion by inadvertently pulling the hand driver toward their body, increasing the risk of root contact. To avoid this, the clinician may consider using a finger wrench or work the hand driver slightly away from their body with each turn. If the miniscrew begins to approximate the periodontal ligament, the patient will experience increased sensation under topical anesthesia [69, 74]. If root contact occurs, the miniscrew may either stop or begin to require greater insertion strength. If trauma is suspected, the clinician should unscrew the miniscrew 2 or 3 turns and evaluate it radiographically.

### 5.2. Stationary Anchorage Failure under Orthodontic Loading

According to the literature, the rates of stationary anchorage failure of miniscrews under orthodontic loading vary between 11% and 30% [75–77]. If a miniscrew loosens, it will not regain stability and will probably need to be removed and replaced [59]. Stability of the orthodontic miniscrew throughout treatment depends on bone density, peri-implant soft tissues, miniscrew design, surgical technique, and force load [78–82]. The key determinant for stationary anchorage is bone density [83, 84]. Stationary anchorage failure is often a result of low bone density due to inadequate cortical thickness [67].

In general, stationary anchorage failure is greater in the maxilla, with the exception of the midpalatal region, due to the greater trabeculae and lower bone density [85, 86]. Loss of midpalat miniscrews is likely a result of tongue pressure. Peri-implant soft tissue type, health, and thickness can affect stationary anchorage of the miniscrew. Miniscrews placed in nonkeratinized alveolar tissues have greater failure rates than those in attached tissues [77]. The movable, nonkeratinized alveolar mucosa is easily irritated; soft tissue inflammation around the miniscrew is directly associated with increased mobility [79]. Additionally, miniscrews placed in regions of thick keratinized tissue, such as the palatal slope, are less likely to obtain adequate bony stability [87]. Thin, keratinized tissue, seen in the dentoalveolar or midpalatal region, is ideal for miniscrew placement [87]. Miniscrew geometry and surgical technique directly influence the stress distribution of peri-implant bone [78]. Most miniscrew losses occur as a result of excessive stress at the screw-bone interface [75]. Self-drilling miniscrews can have greater screw-bone contacts (mechanical grip) and holding strengths compared with self-tapping screws [86–88]. Heidemann et al. [87] reported greater residual bone between screw threads of self-drilling miniscrews compared with self-tapping miniscrews. Self-tapping miniscrews, like self-drilling screws, can be placed without a predrilled pilot hole in the dentoalveolar region if the cortical bone is thin [89]. If a pilot hole is to be used, for either self-drilling or self-tapping miniscrews, the pilot hole size should be no greater than 85% of the diameter of the miniscrew shaft for optimal stability [90]. It is still not clear the maximum force-load, a miniscrew can withstand with regard to stationary anchorage [86]. Dalstra et al. [91] reported that miniscrews inserted into thin cortical bone and fine trabeculae should be limited to 50 g of immediate loaded force. Büchter et al. [76] reported that miniscrews placed in dense mandibular bone remained clinically stable with up to 900 g of force. Many articles reported miniscrew stability with loading forces of 300 g or less [76, 81, 92]. In regions of poor bone density, simply placing a longer miniscrew under smaller orthodontic force does not ensure stationary anchorage [93]. 

### 5.3. Soft Tissue Coverage of the Miniscrew Head and Auxiliary

Miniscrews placed in alveolar mucosa, particularly in the mandible, might become covered by soft tissue. The bunching and rubbing of loose alveolar tissue can lead to coverage of both the miniscrew head and its attachments (i.e., coil spring, elastic chain) within a day after placement. Soft tissue coverage might be a risk factor for miniscrew stability, as well as a clinical concern for the patient, who might think that the miniscrew has fallen out. Miniscrew attachments (elastic chain, coil spring) that rest on tissues will likely become covered by tissue. The soft tissue overlaying the miniscrew is relatively thin and can be exposed with light finger pressure, typically without an incision or local anesthetic. Soft tissue overgrowth can be minimized by placement of a healing abutment cap, a wax pellet, or an elastic separator [94]. In addition to its antibacterial properties that minimize tissue inflammation, chlorhexidine slows down epithelialization and might reduce the likelihood of soft tissue overgrowth [95]. The authors suggest partial insertion with a longer miniscrew (10 mm) in regions of loose alveolar mucosa, leaving 2 or 3 threads of the shaft exposed to minimize the possibility of soft tissue coverage.

### 5.4. Soft Tissue Inflammation, Infection, and Peri-Implantitis

Healthy peri-implant tissue plays an important role as a biologic barrier to bacteria [96]. Tissue inflammation, minor infection, and peri-implantitis can occur after miniscrew placement [97]. Inflammation of the peri-implant soft tissue has been associated with a 30% increase in failure rate [79]. Peri-implantitis is inflammation of the surrounding implant mucosa with clinically and radiographically evident loss of bony support, bleeding on probing, suppuration, epithelia infiltrations, and progressive mobility [95]. The clinician should be forewarned of soft tissue irritation if the soft tissues begin twisting around the miniscrew shaft during placement. Some clinicians advocate a 2-week soft tissue healing period for miniscrews placed in the alveolar mucosa before orthodontic loading [98].

## 6. Feature of Mini-Implants

The rates of stationary anchorage failure of miniscrews under orthodontic loading vary between 11% and 30% [75–77]. The rates are not low, and it may leave much room for improvement. However, the improvement in designed screw including the diameter, length, and thread may reach the limit. Therefore, new materials instead of titanium such as CaP may be necessary to investigate in future.

## 7. Conclusions

On the basis of this systematic review, the following can be concluded.

1. Miniscrew implants can function as viable alternative to conventional molar anchorage. They are simple and efficient anchors for canine retraction, especially in moderate to maximum anchorage situations.
2. The placement of mini-implants for skeletal anchorage may provide the intrusion of posterior without the side effect of extrusion of the anchorage teeth.
